# Optimisation of bacterial release from a stable microfluidic-generated water-in-oil-in-water emulsion†

**DOI:** 10.1039/d0ra10954a

**Published:** 2021-02-17

**Authors:** Nur Suaidah Mohd Isa, Hani El Kadri, Daniele Vigolo, Konstantinos Gkatzionis

**Affiliations:** Faculty of Fisheries and Food Science, Universiti Malaysia Terengganu 21030 Kuala Terengganu Terengganu Malaysia; School of Chemical Engineering, University of Birmingham Birmingham B15 2TT UK; School of Biomedical Engineering, University of Sydney NSW 2006 Australia daniele.vigolo@sydney.edu.au; Department of Food Science and Nutrition, School of the Environment, University of the Aegean Metropolite Ioakeim 2 81400 Myrina Lemnos Greece kgkatzionis@aegean.gr

## Abstract

Application of droplet microfluidics for the encapsulation of bacteria in water-in-oil-in-water (W/O/W) emulsion allows for production of monodisperse droplets with controllable size. In this study the release of bacteria from W/O/W emulsion, the effect of the double emulsion structure on bacterial growth and metabolic activity, and the stability and mechanism of bacterial release were investigated. W/O/W emulsions were formed using a double flow-focusing junction microfluidic device under controlled pressure to produce droplets of approximately 100 μm in diameter containing an inner aqueous phase (W_1_) of about 40–50 μm in diameter. GFP-labelled *Escherichia coli* (*E. coli*-GFP) bacteria were encapsulated within the W_1_ droplets and the stability of emulsions was studied by monitoring droplet size and creaming behaviour. The double emulsions were stabilised using a hydrophilic (Tween 80) and a lipophilic surfactant (polyglycerol polyricinoleate) and were destabilised by altering the osmotic balance, adding NaCl either in the inner W_1_ phase (hypo-osmotic) or outer W_2_ phase (hyper-osmotic). The release of *E. coli*-GFP was monitored by plating on agar whereby the colony form unit (CFU) of the released bacteria was determined while fluorescent microscopy was employed to observe the mechanism of release from the droplets. The release of *E. coli*-GFP was significantly increased with higher concentrations of NaCl and lower amounts of Tween 80. Microscopic observation revealed a two-step mechanism for the release of bacteria: double W/O/W emulsion droplet splitting to release W_1_ droplets forming a secondary double emulsion followed by the collapse of W_1_ droplets to release *E. coli*-GFP into the continuous aqueous phase.

## Abstract 2

Encapsulation enhanced viability and metabolic activity. Nutrients can cross the oil layer. Bacterial release increased while emulsion stability decreased at high osmotic pressure and low surfactant concentration. Two-step release mechanism observed.

## Graphical abstract

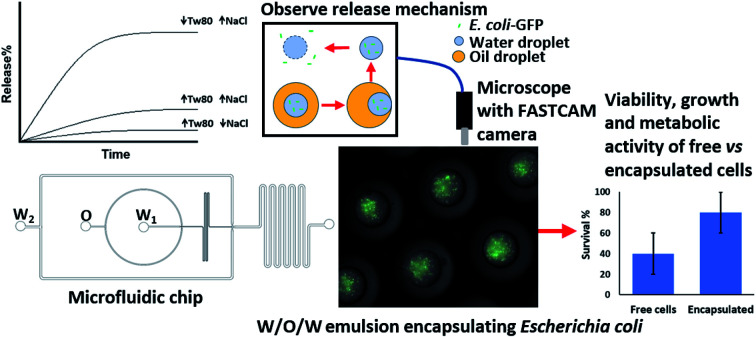

## 1. Introduction

A double water-in-oil-in-water (W/O/W) emulsion is an emulsion within an emulsion where two aqueous phases, namely the inner (W_1_) and outer (W_2_) phases are separated by an immiscible layer of oil (O) to form smaller droplets that are encapsulated within a larger droplet. The uses of double W/O/W emulsions extend to various areas, from lab-scale studies to industrial applications.^1^ Double emulsions have been extensively used in pharmaceuticals for drug delivery^2^ and cosmetics.^3^ In food applications, they have been used to produce reduced-fat products, encapsulate and protect sensitive compounds such as bioactive and natural colouring.^4^ This approach also offers an advantage over other encapsulation methods by allowing the encapsulation of hydrophilic substances whilst reducing its calorific value without compromising its textural and sensorial properties.^5^ Depending on their application, W/O/W emulsions can be produced in a controlled manner with a single inner droplet core^6^ or with multiple inner droplets for compartmentalization.^7^

Microbial encapsulation in double W/O/W emulsion has led to various advancements in the study of complex bacterial interactions,^8^ to protect probiotics against harsh processing and gastrointestinal conditions,^5,9–11^ segregate different species during fermentative processes,^12,13^ triggered-release of bacterial cells in a controlled-manner,^14,15^ understand biological activities such as genetic activation, quorum sensing^8^ and development of biofilms.^16^ Additionally, there has been an increased interest in the application of the droplet microfluidic technique for studying bacterial encapsulation in W/O/W emulsion due to its advantages compared to conventional techniques such as homogeneity of droplet size and precise tailoring of physicochemical conditions. Furthermore, encapsulation of bacteria in double emulsions generated by microfluidics has the advantage over other methods in that it can precisely control the number and species of bacteria being encapsulated in each W_1_ droplet.

It has been reported previously that the controlled release of hydrophilic materials/cargo entrapped within W/O/W emulsion can be induced by several factors such as osmotic imbalance,^7,14,17^ change in pH,^18,19^ and alteration in temperature.^19,20^ Triggered release of *Escherichia coli* was achieved in W/O/W emulsion by altering the osmotic balance between inner W_1_ and outer W_2_ aqueous phases through the addition of NaCl.^14,15^ Moreover, the rate of bacterial release depends on the osmotic pressure between W_1_ and W_2_ phases, concentration of hydrophilic and lipophilic surfactants and volume of W_1_ phase. It was concluded that the osmotic balance alteration leads to droplets bursting, resulting in the release of *E. coli* into the W_2_ phase.^14,15^ However, W/O/W emulsions prepared by two-stage homogenization produces droplets with multiple inner aqueous phase of uneven numbers and droplets with polydisperse size. A better control on the size of droplets is required for an in depth understanding of microbial release from W/O/W emulsion that can be achieved by droplet microfluidic.^8,16,21,22^

The effect of immobilisation of *E. coli* cells have been described in previous studies including an increase in the metabolic activity of *E. coli* cells due to their adhesion onto a glass surface,^23^ an increase in oxidized glucose metabolites^24^ and the entrapment of *E. coli* cells that resulted in better enzymatic activity and reduced degradation of RNA.^25^ The change in metabolic activity may be attributed to the change in microenvironment conditions such as a reduction in the water activity and oxygen supply which usually occurs in an immobilized microenvironment.^24^ These changes not only affect bacterial activity but also make them less susceptible to environmental stresses. The compartmentalizing structure of W/O/W emulsion can affect microbial cells. For example, W/O/W emulsion was used to segregate *Zygosaccharomyces rouxii* (in W_1_) and *Tetragenococcus halophilus* (in W_2_), two predominant microbial species in soy sauce fermentation.^12,13^ It was shown that delivering the mixed cultures of *T. halophilus* and *Z. rouxii* in reduced-salt moromi could compensate for the changes in the final overall aroma balance of high salt concentration by promoting the formation of some essential volatile compounds resulting in a product with volatile profile pattern identical to the original high-salt soy sauce.^13^ Encapsulation in double W/O/W emulsion has shown to enhance (increase of 3 log CFU mL^−1^) the survival of *Z. rouxii* during a 30 day storage.^12^ It was suggested that the oil layer acts as a barrier and can reduce mass transfer and communication between cells in W_1_ and W_2_ phases causing them to enter a non-dividing resting state and thus become more resistant to environmental stresses.

Nutrient consumption and metabolic waste secretion within the droplets may cause instability as it changes the composition of the droplets. The metabolic activity of bacteria within the droplets may lead to continuous change in solute concentration due to nutrient depletion and the production of metabolic by-products. In W/O emulsions, *E. coli* metabolise nutrients and excrete waste that diffuse through the oil layer and into the surrounding droplets altering the osmotic balance and causing diffusion of solutes and droplet shrinkage at rates depending on the strain, species and bacterial cell concentration.^26^

The stability of double emulsions, which is essential for practical applications, remains a challenge due to their inherent thermodynamic instability. Moreover, this study investigates the stability of double emulsions in the presence of bacteria during growth, metabolic activity and release which are all important events that may alter the composition of the aqueous phases (*e.g.* osmotic balance alterations) and therefore affect the double emulsion's stability. A better understanding on the stability of double emulsions encapsulating bacteria allows for tailoring the emulsion structure to extend the duration of their stability during applications. In contrary, destabilisation of double emulsion structure may be desirable in certain applications which if controlled can allow for triggered release of bacteria in a controlled manner. In this study, the effect of bacterial encapsulation in double W/O/W emulsions on droplets stability and bacterial viability were investigated with the application of droplet microfluidic to ensure precise control over the encapsulation process. Non-ionic surfactants such as polyglycerol polyricinoleate (PGPR) and polysorbate 80 (Tween 80) were used as they are less likely to interact with bacterial cells.^27^

## 2. Materials and methods

### 2.1 Materials and bacterial cultures

Microfluidic device fabrication was done by using a polydimethylsiloxane (PDMS) preparation set (Sylgard 184, Dow-corning, United States). The oil-soluble surfactant, polyglycerol polyricinoleate (PGPR) was obtained from Danisco (Denmark). Mineral oil, sodium chloride (NaCl) were from Fischer Scientific (United Kingdom) while Poly(allylamine hydrochloride) (*M*_w_ ≈ 17 500), poly(sodium 4-styrenesulfonate) (*M*_w_ ≈ 70 000) and water-soluble surfactant, polysorbate 80 (Tween 80) were purchased from Sigma Aldrich (United Kingdom). For bacterial study, the material used were nutrient agar, Luria Bertani broth (LB broth), tryptone, yeast extract and phosphate buffer saline (PBS) all by Oxoid Ltd. (United Kingdom). d(+)-glucose was purchased from Acros Organics (United Kingdom) and Nile red stain was purchased from Invitrogen™ (United Kingdom). *Escherichia coli* strain SCC1 (MG1655-GFP mutation) expressing green fluorescent protein (*E. coli*-GFP) stock culture was obtained from Biochemical Engineering Laboratory, University of Birmingham, United Kingdom.

### 2.2 Microfluidic device fabrication

**Fig. 1:**
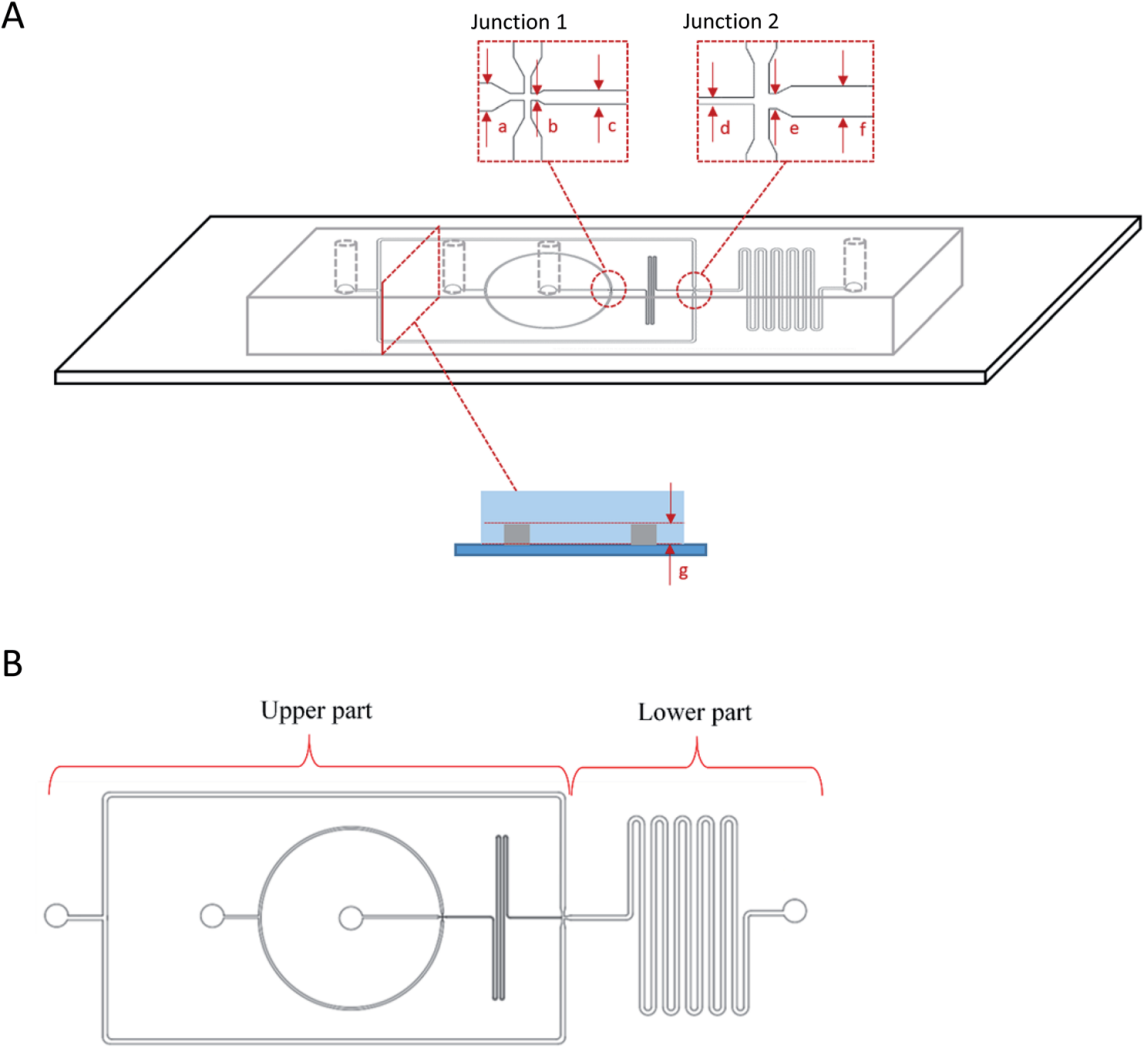
Flow-focusing microfluidic device side (A) and top (B) view showing the two junctions for one-step W_1_/O/W_2_ droplets generation. W/O droplets were first produced at junction 1 that were then flowed into junction 2 for further encapsulation into the second aqueous phase (W_2_) forming W_1_/O/W_2_ droplet. The dimensions of the microfluidic device were, (a) 100 μm, (b) 25 μm, (c) 50 μm, (d) 50 μm, (e) 100 μm, (f) 200 μm for the widths of the channels while the depth of the channel was (g) 50 μm.

A device with two flow-focusing junctions was used for one-step production of double W/O/W droplet (Fig. 1). The device was made by using standard soft lithography technique^28^ and was designed according to Bauer *et al.*^29^ (2010) by using AutoCAD 2016 (Autodesk) software. The design was then printed onto high-resolution photomasks (Micro Lithography Services Limited, UK) and a patterned mould was produced by exposing a silicon wafer (Si-Mat, Germany) that was spin-coated with SU-8 photoresist (SU-8, Microchem) to UV light through the photomasks with a mask aligner (Canon PLA-501FA mask aligner). The device was then prepared by mixing the PDMS and curing agent at the recommended mixing ratio of 1 : 10. The prepared PDMS was then poured onto the mould, degassed, and cured in an oven at 70 °C for 1 hour. The device was then cut out of the mould and the inlet and outlet holes were punched followed by corona discharge treatment (Relyon, PZ2) for approximately 30 s that bonds the device onto a glass slide to close the channels. The prepared device was then left on a hot plate for approximately 15 min at 100 °C. A new device was prepared for every experiment in order to minimize contamination.

The production of double W/O/W emulsion droplet requires partial surface modification of the microfluidic device. The prepared PDMS device was partially surface-treated.^29^ The process was conducted by loading a polyelectrolyte multilayer (PEM) sequence composed of poly(allylamine hydrochloride) (PAH) and poly(sodium 4-styrenesulfonate) (PSS) solutions in 0.5 M aqueous sodium chloride solution (0.1% w/v) with sodium chloride (NaCl) in deionized water (0.1 M) as washing solution into a Tygon tube (Cole-Parmer instrument Co. Ltd., UK). The PEM sequence was then flushed through the lower part of the device by using a syringe pump while blocking the upper part with a stream of deionised water through. At the end of the process, the lower part of the microfluidic device was coated with a sequence of PAH–PSS–PAH–PSS–PAH–PSS polyelectrolyte multilayer (PEM) rendering hydrophilic channel walls. The upper part of the microfluidic device was left untreated and was allowed to recover the typical hydrophobicity of PDMS for 24 hours before use in order to ease the production of W/O droplets at the first junction (Fig. 1).

### 2.3 Bacterial cell preparation

*E. coli*-GFP for encapsulation in W/O/W emulsion was cultured on nutrient agar at 37 °C for 24 hours. The bacterial cells were inoculated into 50 mL of Luria Bertani broth (LB Broth) in a shaking incubator at 37 °C, 150 rpm for 24 hours and sub-cultured into LB broth (1 : 50) and incubated for another 2 hours. The culture was then centrifuged (10000 g, 10 min) and washed twice with 50 mL of PBS. After centrifugation, the supernatant was replaced with 50 mL of fresh LB broth or deionized water (DIW) to re-suspend bacterial cells for encapsulation. The cell concentration was prepared to 10^8^ CFU mL^−1^.

### 2.4 Microfluidic encapsulation of *E. coli*-GFP in double W/O/W droplet

Culture of *E. coli*-GFP in LB broth or DIW was used as the inner aqueous phase of W/O/W emulsion droplet. The oil phase consisted of mineral oil with 1.5% w/v PGPR surfactant while W_2_ phase consisted of either LB broth or DIW with 1% w/v Tween 80 surfactant for samples used in the viability study. For the study that determined the effect of osmotic balance alterations on bacterial release and droplet stability, sodium chloride (NaCl) was added to either the inner W_1_ phase (hypo-osmotic) or outer W_2_ phase (hyper-osmotic) at 0.5%, 1.0% or 1.5% w/v. Tween 80 was added in the W_2_ phase at 1% or 5% w/v.

The double junction flow-focusing microfluidic device was connected to a pressure controller (OB1 MK3, Elveflow, France) to produce monodispersed droplet of approximately 40–50 μm in diameter for W_1_ phase and approximately 100 μm in diameter for the oil globule (ESI Fig. S1B†). The produced W/O/W droplet were collected continuously at the outlet. Droplet formation was monitored by using a microscope (Nikon Ti-U, Japan) equipped with a fast camera (Photron FASTCAM S5) (ESI Fig. S1A†). The pressures used were 300 mbar for the internal aqueous phase containing *E. coli*-GFP cells, 310 mbar for the middle oil phase and 330 mbar for the outer aqueous phase. W/O/W emulsion free of cells were also produced as controls. All samples were kept statically in Eppendorf tubes at 25 °C.

### 2.5 Determining bacterial viability in different formulations of W/O/W emulsions

The viability of *E. coli*-GFP cells were determined for cells encapsulated in different formulations of W/O/W emulsions. Non-encapsulated bacteria dispersed in DIW or LB broth were prepared as controls. Encapsulated samples were centrifuged at 15800 g for 10 min in order to break the emulsion and release the entrapped cells. Serial dilutions of the sample were done using phosphate buffer saline (PBS) and viable cells were counted overtime for 24 hours using the Miles and Misra method.^30^

### 2.6 Changes in cell metabolic activity

In order to determine the effect of encapsulation on the metabolic activity of bacteria, the change in glucose concentration in the presence of encapsulated and free *E. coli*-GFP cells was measured over time for 24 hours. Samples of encapsulated *E. coli*-GFP in DIW were prepared along with samples of free *E. coli*-GFP cells suspended in DIW as non-encapsulated control. Non-bacterial controls were also prepared that included a sample with empty W/O/W emulsion droplet. Approximately 2 mL of the prepared samples were transferred into LB broth supplemented with 0.6% w/v glucose and the change in glucose concentration was measured by using Accu-chek Aviva monitor with Accu-chek Aviva glucose test strips from Roche diagnostics (United Kingdom). The test was done according to the manufacturer instructions.

### 2.7 Fluorescence microscopy for bacterial observation

Fluorescence microscopy was performed at room temperature for *E. coli*-GFP observation in W/O/W emulsion droplets and to study the release of bacteria. The sample was prepared for microscopy by placing a drop of sample onto the glass slide and covered with a coverslip. In order to track the middle oil phase, the oil phase was stained with Nile red prior to sample preparation. The sample was then observed at 40× magnification and micrographs of the samples were acquired by using a Zeiss Axioplan microscope equipped with a 10-megapixel CMOS Motic Moticam digital colour camera system and Motic images plus software. The emission was observed at 509 nm for GFP and 640 nm for Nile red.

### 2.8 Measuring the encapsulation efficiency and release of bacteria from W/O/W emulsion droplets

Encapsulation efficiency and the release of bacteria was determined by measuring the number of cells in the outer aqueous phase immediately after droplet preparation and over time for 3 hours. Samples were collected in Eppendorf tubes and kept statically upright at 25 °C causing creaming or phase separation that divided the samples into a cream layer (oil globules) and serum phase (W_2_) due to the difference in density between the two phases. Approximately 1 mL of serum was carefully withdrawn from the sample by using a pipette and serially diluted in PBS. Cell counts were done by plating on nutrient agar through Miles and Misra method. As the serum phase does not contain oil globule, unencapsulated and released cells could be quantified according to the method described by El Kadri *et al.*^14^ (2015) with the following equation:Encapsulation efficiency (%) = ((*N*_0_ − *N*)/*N*_0_) × 100Bacterial release = log_10_ *N* − log_10_ *N*_*T*_where *N* is the viable cell count for unencapsulated bacteria in W_2_ immediately after emulsion preparation and *N*_0_ is the total number of viable cell count before encapsulation into W/O/W emulsion. *N*_*T*_ is the number of viable cells in the W_2_ phase at incubation time, *T*.

### 2.9 Determination of droplet size and phase separation

Changes in the diameter of the W_1_ phase and the oil globule was measured overtime for 3 hours at 25 °C. In order to measure the size of the droplets, microscopy observation was conducted by placing the sample on a glass slide and observed at 10× magnification using a Nikon Eclipse Ti-U microscope. Images of the droplets were taken and analysed by MATLAB software using the circular Hough transform.^31^

Droplet stability was also determined by measuring changes in creaming behaviour of the W/O/W emulsion with or without the presence of *E. coli*-GFP. Creaming behaviour was measured by collecting 1 mL of sample into graduated syringes immediately after emulsion preparation. The samples were left upright for 1 hour at 25 °C to allow the formation of a cream layer. The cream thickness was then measured immediately and during incubation time at 30, 60 and 180 minutes. The creaming volume was calculated as follows:
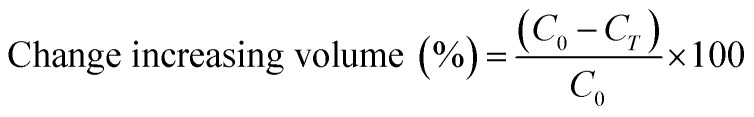
where *C*_0_ is the creaming height at time 0 while *C*_*T*_ is the creaming height at incubation time, *T*.

### 2.10 Statistical analysis

The experiments were conducted with three replicates. The generated data were analysed with Excel (Microsoft Corp.) in order to calculate the mean, standard deviation (SD), standard error of mean (SEM) values. One-way ANOVA with Tukey's HSD was conducted to compare several means by using IBM SPSS statistical software version 23. The difference between the means was considered significant at *P* < 0.05.

## 3. Results and discussion

### 3.1 Encapsulation efficiency, viability, and metabolic activity of bacteria in W/O/W emulsion droplets

**Fig. 2:**
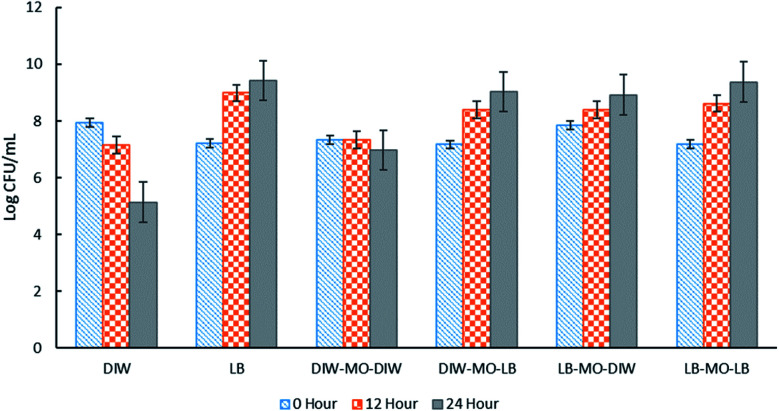
The effect of different W_1_/O/W_2_ emulsion formulations on the viability of *E. coli*-GFP cells. Free cells in DIW and LB broth were tested as controls against samples of bacteria encapsulated in W_1_/O/W_2_ emulsion. Log CFU mL^−1^ of the samples were determined at 0, 12 and 24 hours of incubation. Bars represent mean ± SEM taken from 3 independent experiments with 3 replicates for each experiment (*N* = 3). Abbreviations, DIW: deionised water, MO: mineral oil with 1.5% PGPR, LB: LB broth.

**Fig. 3:**
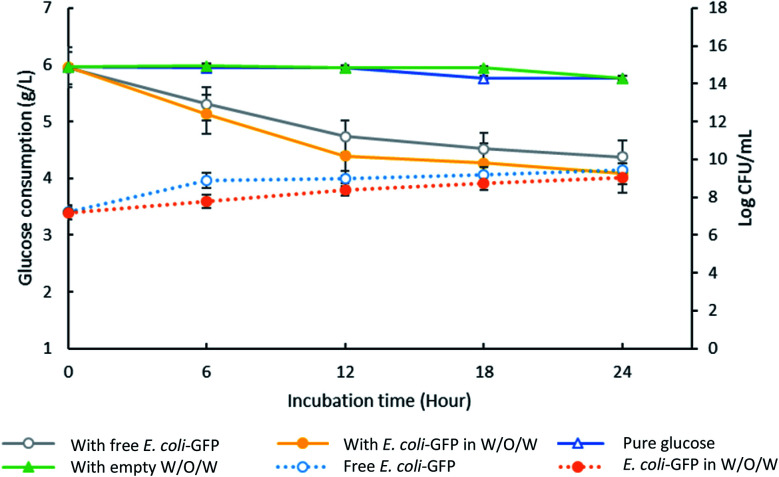
Changes in glucose concentration in the presence of encapsulated and free *E. coli*-GFP cells. The solid lines represent data for glucose consumption for samples containing free *E. coli*-GFP and encapsulated *E. coli*-GFP in W/O/W emulsion against control samples containing empty W/O/W emulsion and pure glucose (without the addition of free bacteria, encapsulated bacteria or empty W/O/W). The dotted lines represent changes in log CFU ml^−1^ for free (unencapsulated) bacterial cells and encapsulated cells in W/O/W emulsion. All of the prepared samples are transferred into LB broth supplemented with 0.6% w/v glucose. Bars represent mean ± SEM taken from 3 independent experiments with 3 replicates for each experiment (*N* = 3).

High encapsulation efficiency of approximately 99.9% of *E. coli*-GFP viable cells in W/O/W emulsion droplets was achieved (Table S1†). In order to understand the effect of encapsulation on the growth and metabolic activity of cells, the viability (Fig. 2) and rate of glucose consumption (Fig. 3) were monitored over time.

As expected, the absence of LB broth caused a reduction in bacterial viability of both encapsulated and free *E. coli*-GFP cells (Fig. 2). A greater reduction in bacterial viability was observed for free cells as compared to encapsulated cells. In the absence of nutrients (*E. coli*-GFP in DIW only), the viable cell count was reduced from 7.94 to 5.13 log CFU mL^−1^ after 24 hours (2.81 log CFU mL^−1^ reduction) while for encapsulated cells (DIW-MO-DIW), the cell count was reduced from 7.33 to 6.97 log CFU mL^−1^ (0.33 log CFU mL^−1^ reduction) after 24 hours. The ability of W/O/W emulsion to improve the viability of bacterial cells during storage, processing and against harsh processing conditions has been reported previously in various studies.^5,9,10,12,32^ The protective effect of W/O/W emulsion is mainly caused by its role as a buffer against environmental conditions.^5^

In the presence of LB broth, the log CFU mL^−1^ of *E. coli*-GFP increased over time for all W/O/W emulsion formulations (Fig. 2). Higher growth was observed for free *E. coli*-GFP cells suspended in LB broth compared to cells encapsulated in the presence of LB broth in both or either W_1_ or W_2_ phase. Samples prepared with LB broth in W_2_ showed a higher increase in log CFU mL^−1^ compared to samples with LB broth in W_1_. Moreover, samples prepared with LB in W_2_ and DIW in W_1_ showed a similar growth pattern with samples prepared with LB in both W_1_ and W_2_ phases which indicates that nutrients can cross from W_2_ through the oil layer barrier to reach W_1_. The encapsulation of bacteria in W/O/W emulsion droplets allows a continuous supply of nutrients from the outer aqueous phase to the inner phase. It has been reported previously that the oil phase of the W/O/W emulsion acts as a selective barrier that modulates the transport of molecules, allowing the diffusion of nutrients and small inducer molecules through the interface.^8,33,34^ This creates a controlled microenvironment whereby W/O/W emulsion droplets serve as micro-incubators that sustain the growth of bacteria. Furthermore, the immobilization of bacteria in W/O/W emulsion droplet may cause morphological and physiological changes which may play a role in their survival.^24^

Although free *E. coli*-GFP showed higher viability, the reduction in glucose concentration was faster for samples containing encapsulated cells (Fig. 3). After 24 hours, samples containing encapsulated and free *E. coli*-GFP cells showed 31% and 26% reduction in glucose concentration respectively. Encapsulation of *Z. rouxii* in W/O/W emulsion has also been reported previously that alter the metabolic activity and accelerate glucose consumption.^13^

### 3.2 The release of *E. coli*-GFP from W/O/W emulsions

The presence of solutes in W_1_ and W_2_ phases can alter the osmotic balance and the emulsion's stability. Therefore, it is important to understand its effect towards droplet stability and for application of W/O/W in the controlled release of bacteria. For this study, it is crucial to ensure that the solute used is not able to support bacterial growth as it may affect the accuracy of the results. LB broth consists of tryptone, yeast extract and NaCl. Each of these ingredients was tested for its ability to support bacterial growth (ESI Table S2†). NaCl was found to be a suitable solute to test the osmotic effect due to its incapability to support growth. The effect of NaCl addition in either W_1_ or W_2_ phase was studied to create hypo-osmotic (NaCl in W_1_) or hyper-osmotic (NaCl in W_2_) conditions, respectively. The NaCl concentrations that were tested in this study were 0.5% w/v (similar to that found in LB broth), 1.5% w/v and 2.0% w/v based on the bacterial tolerance towards NaCl as observed in previous studies.^35^

**Fig. 4:**
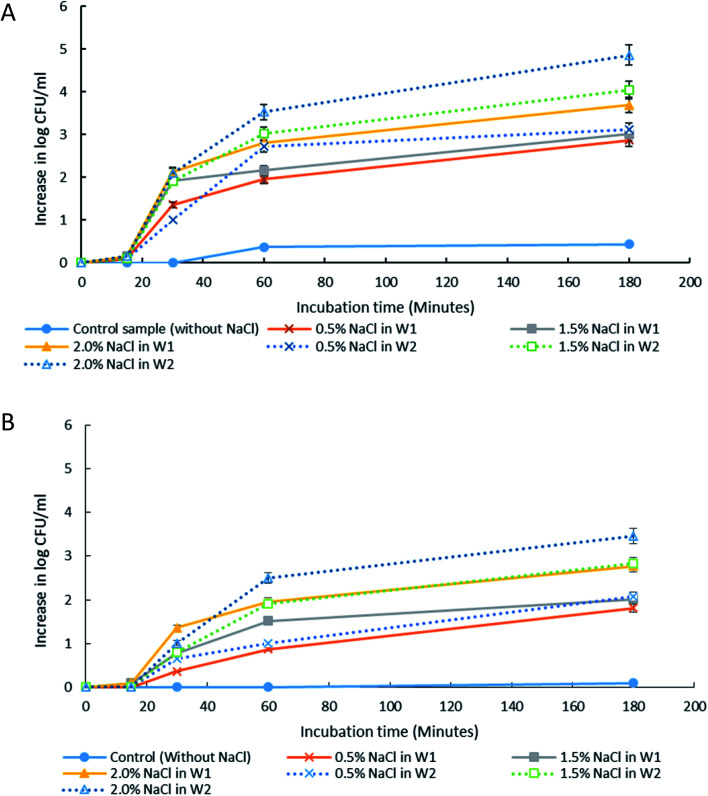
The release of *E. coli*-GFP into the outer aqueous phase (W_2_) of W/O/W emulsion droplet at 15, 30, 60 and 180 minutes of incubation at 25 °C. Samples were prepared with different NaCl concentration in either inner (W_1_) or outer aqueous phase (W_2_). Tween 80 concentration in W_2_ was also differentiated with (A) 1% w/v and (B) 5% w/v. Bars represent mean ± SEM taken from 3 independent experiments with 3 replicates for each experiment (*N* = 3).

**Fig. 5:**
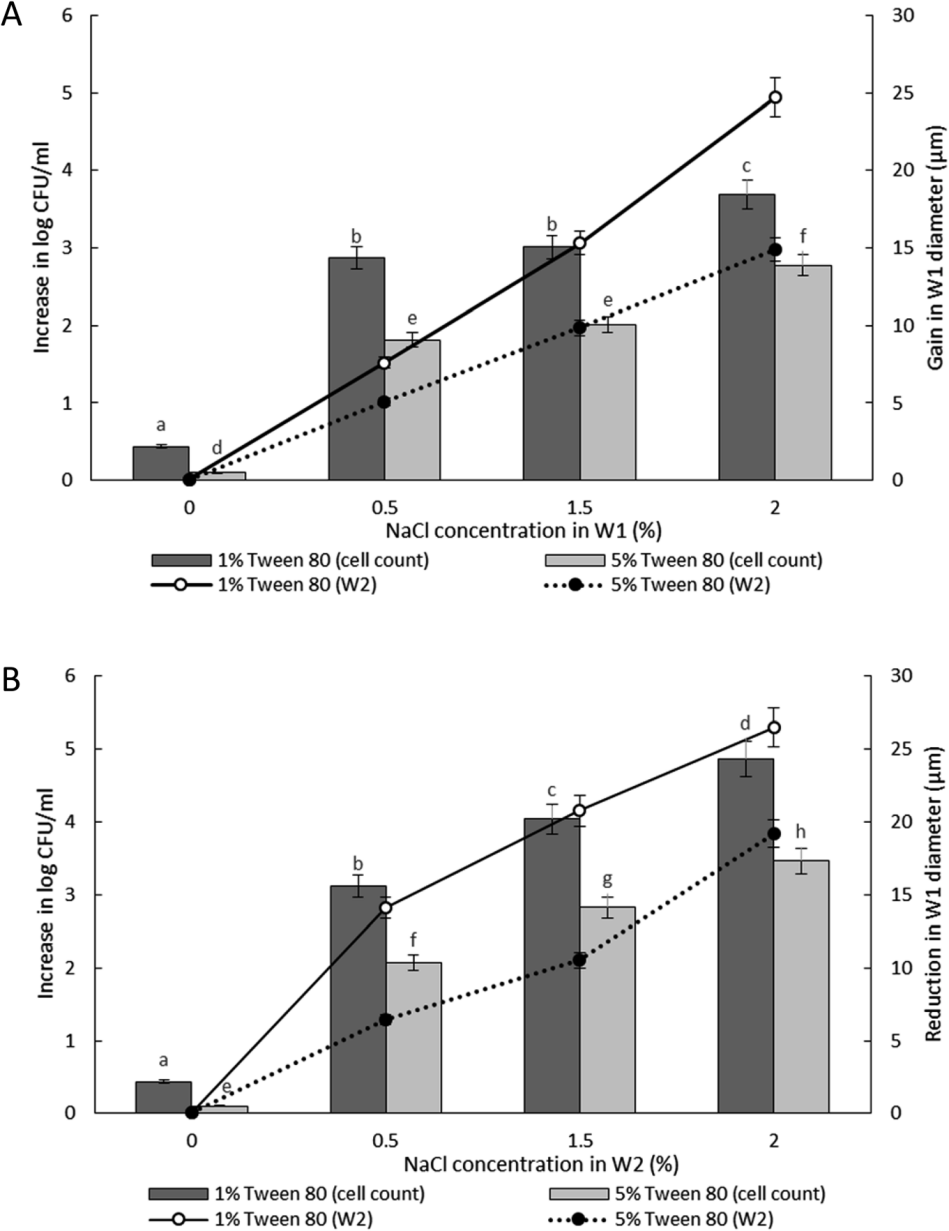
Increase in viable cell count and changes in droplet diameter with different NaCl concentration of 0%, 0.5%, 1.5% and 2.0% w/v in inner W_1_ phase (A) and outer W_2_ phase (B) of W/O/W emulsion droplet after 180 minutes of incubation. Bar chart represents the data for the increase in viable cell count while the line graph represents the data for changes in droplet diameter. Bars represent mean ± SEM taken from 3 independent experiments with 3 replicates for each experiment (*N* = 3). The data for the increase in viable cell count was analysed with one-way ANOVA. ^a b c d e f g h^ mean ± SEM with different letters are significantly different at *P* < 0.05.

The release of encapsulated *E. coli*-GFP cells from W/O/W emulsions with different NaCl concentrations are presented in Fig. 4 and 5. It was observed that osmotic balance alterations resulted in a significant increase (*P* < 0.001) in release of *E. coli*-GFP cells. No significant increase in bacterial release was observed for samples without NaCl during 180 minutes of incubation period for both samples prepared with 1% and 5% of Tween 80 in the W_2_ phase. The addition of NaCl in the W_1_ phase (hypo-osmotic) and W_2_ phase (hyper-osmotic) resulted in a gradual release of bacteria which begins after 30 minutes of incubation followed by a complete bacterial release after 180 minutes of incubation. The rate of release of *E. coli*-GFP cells depends on NaCl concentration in W_1_ and W_2_ phases whereby a higher increase in release of *E. coli*-GFP cells occurred at higher NaCl concentration (Fig. 4). In addition, a higher release of *E. coli*-GFP cells was also observed under hyper-osmotic as compared to hypo-osmotic condition throughout the incubation period. These results are in agreement with previous work showing that bacterial release increased with increase in NaCl concentration during hypo-osmotic^14^ and hyper-osmotic^15^ alterations.

The release of encapsulated *E. coli*-GFP cells from W/O/W emulsions prepared with different Tween 80 concentrations (1% or 5%) in the W_2_ phase are presented in Fig. 5. The release of *E. coli*-GFP cells was significantly (*P* < 0.05) higher with higher amounts of NaCl and lower Tween 80 concentration in both hypo-osmotic and hyper-osmotic conditions. These results are in agreement with previous studies.^14,15^ Moreover, the release of *E. coli*-GFP was ∼1 log CFU mL^−1^ (with 1% Tween 80) and ∼0.5 log CFU mL^−1^ (with 5% Tween 80) higher under hyper-osmotic (Fig. 5B) as compared to hypo-osmotic (Fig. 5A) conditions at higher amounts of NaCl (1.5 and 2% NaCl).

**Fig. 6:**
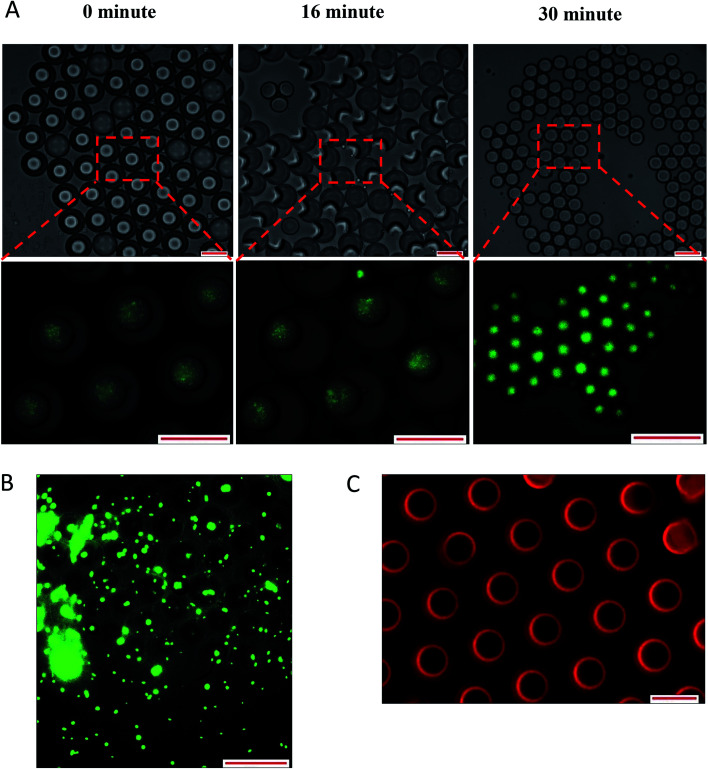
Optical and fluorescence photomicrographs of droplet splitting releasing inner W_1_ phase (A) leading to release of bacteria from the released inner W_1_ droplet after 180 minutes of incubation (B). The oil phase was stained with Nile red in order to distinguish between the aqueous and the oil phase (C). Droplet were prepared with 0.5% NaCl and 1% of Tween 80 in outer W_2_ phase. Scale bar represents 100 μm.

Overall, the instability of W/O/W emulsion induced by osmotic balance alterations led to the release of bacteria as confirmed by microscopic observation immediately after droplet formation (Fig. 6). The oil phase was stained with Nile red in order to distinguish the middle oil phase from the inner and outer aqueous phase (Fig. 6C). Droplets observation reveals the splitting of W/O/W droplet forming a secondary double emulsion containing bacteria enclosed by a very thin film during the first 30 minutes of observation (Fig. 6A). This was followed by changes in droplet size as a function of NaCl concentration that causes swelling of droplet with the addition of NaCl in the inner W_1_ phase or shrinking of droplet with the addition of NaCl in the outer W_2_ phase. Bacterial release occurred only after droplet splitting as shown in Fig. 6A. A significant (*P* < 0.001) increase in release of *E. coli*-GFP was observed after 30 minutes of incubation which occurred immediately after droplet separation. No bacteria were observed in the outer W_2_ phase during droplet splitting which was confirmed by bacterial cell count whereby no increase in log CFU mL^−1^ was observed for all samples during the first 15 minutes of incubation.

The double W/O/W emulsion is metastable and the inner W_1_ phase tends to merge with the continuous outer W_2_ phase forming a more stable O/W emulsion. In a W/O/W emulsion, the difference in density between the oil and the aqueous phase caused the inner W_1_ droplet to move towards the bottom of the oil globule and eventually escape into the outer W_2_ phase forming a small oil globule that moves upwards leaving the inner W_1_ water droplet immersed in the outer W_2_ phase. The escaped water droplet persisted within the outer W_2_ phase and are held by a thin film that consists of a complex mixture of oil and surfactant.^14,36^ The difference in density between the oil and the aqueous phase is presented in Table S3† whereby DIW has a higher density than mineral oil which resulted in the phase separation of the W/O/W droplet.

Jiao, Rhodes and Burgess^37^ (2002) reported on the formation of a dimpled structure caused by the inner droplet that was very close to the interface, separated by only a thin film from the outer W_2_ phase. The formation of the dimpled structure was related to the strength of the thin film surrounding the water droplet in which a film with high elasticity is required to maintain its stability. By increasing the NaCl concentration of the outer W_2_ phase, the interfacial elasticity of the thin film decreases causing droplet instability.^37^ Rapid release of bacteria occurred immediately after the splitting process, therefore, accelerating droplet destabilization. In a study done by Zhang *et al.*^17^ (2017), the intentional destabilization of the droplet by using an ultrathin layer of polymeric shells makes it highly susceptible to osmotic shock due to the rapid diffusion of water into the droplet. Therefore, an immediate burst of droplet releasing encapsulated cargo can be triggered easily by adding a large amount of water in the outer W_2_ phase causing the droplet to swell and burst instantly. Previous studies showed that the release of bacteria in both hyper-osmotic and hypo-osmotic conditions is triggered by the bursting of W/O/W emulsion droplets. Under hypo-osmotic conditions, the swelling of droplets caused the thinning of the interface that resulted in film rupture and release of bacteria into the W_2_ phase.^15^ However, the release of bacteria under hyper-osmotic conditions is not caused by the change in droplet size but to the migration of surfactants desorbing from the interface as water migrates from W_1_ towards W_2_ phase of higher solute concentration.^14^ At a higher surfactant concentration, the replacement of surfactants in the gaps created by the swelling of the droplet under hypo-osmotic conditions and the loss of surfactants from the interface of the droplet under hyper-osmotic conditions is much faster compared to samples with lower surfactant concentration making it much more stable and delays bacterial release.^38^ In addition, higher bacterial release was observed for samples prepared under hyper-osmotic conditions as compared to samples prepared under hypo-osmotic conditions (Fig. 5) which are attributed to the Laplace and osmotic pressure balance. The Laplace pressure (Δ*P*) resulted in the shrinkage of the droplet following:
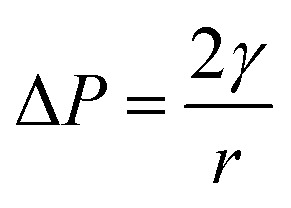
where *γ* the interfacial tension and *r* is the droplet's radius. A smaller radius will cause larger inward force due to Laplace pressure that resulted in droplet shrinkage. However, in the presence of NaCl in the inner W_1_ phase (hypo-osmotic condition), the osmotic pressure resulted in the swelling of the droplet that counterbalanced the Laplace pressure^37^ thus, resulting in a more stable droplets as compared to droplets under hyper-osmotic conditions. The balance between the osmotic and Laplace pressure is given by the Walstra equation:^39^2*γ* = 3*mRT*

In which *m* is the molar concentration of NaCl, *R* is the universal gas constant and *T* is temperature. From this equation, it is shown that optimal NaCl concentration in the internal W_1_ phase is required in order to reach equilibrium. Jiao, Rhodes and Burgess^37^ (2002) reported that the stability of double W/O/W droplet is improved as the salt concentration is closer to the optimal value of 13.4 mol m^−3^. Therefore, emulsions prepared under hypo-osmotic conditions are more stable as compared to emulsions under hyper-osmotic conditions resulting in a lower bacterial release (Fig. 5). As the release of bacteria highly depended on the stability of the droplet, the change in the size of both W_1_ phase and oil globule during osmotic balance alterations was monitored over time in order to further understand its effect on the release of *E. coli*-GFP cells.

### 3.3 The effect of different osmotic conditions on droplet size change

**Fig. 7:**
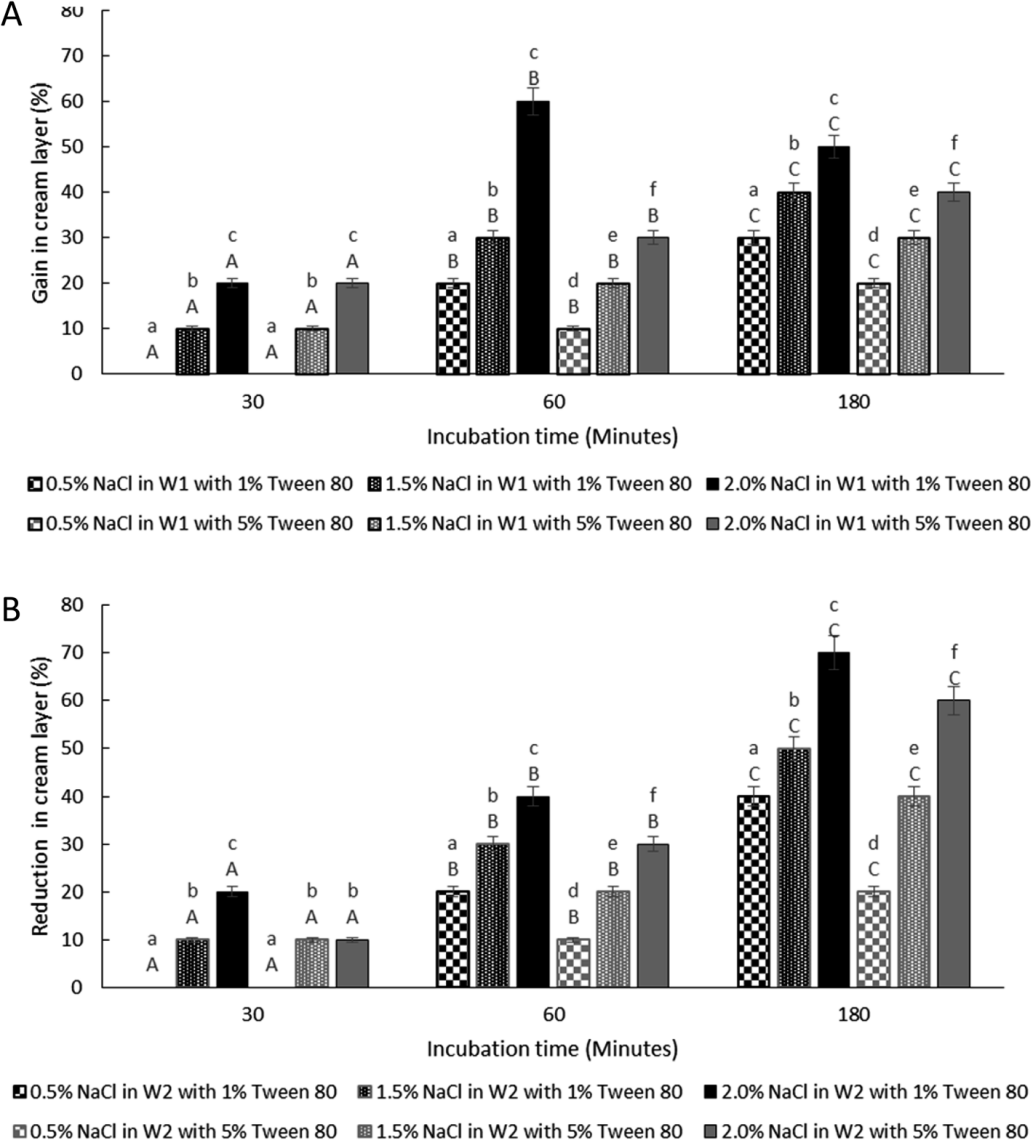
Change in creaming behaviour for samples under hypo-osmotic (A) and hyper-osmotic conditions (B). Formulations were prepared with *E. coli*-GFP in inner W_1_ droplet at different Tween 80 concentration of 1% w/v and 5% w/v along with 1.5% w/v PGPR. Bars represent mean ± SEM taken from 3 independent experiments. Mean comparison with different small and capital letters indicate significant different (*P* < 0.05) between samples within each incubation time and between storage times within each sample, respectively. Data were analysed with one-way ANOVA.

The effect of osmotic balance alterations on the stability of droplet containing bacteria was investigated (Fig. 5 and 7). Photomicrographs of the droplets were taken at every time interval that includes, immediately after droplet preparation (0 minutes), immediately after the release of the W_1_ inner droplet (30 minutes) and followed by 60 and 180 minutes after the separation process. The oil globule phase was distinguished from the inner W_1_ droplet by staining the oil with Nile red prior to the encapsulation process. In addition, the inner W_1_ phase was also identified by the presence of *E. coli*-GFP as the hydrophilic nature of the cells making it more likely to remain in the aqueous phase rather than the oil phase.

Table S4† summarizes the change in the diameter of the inner W_1_ phase and oil globule during the 180 minutes of the incubation period. Osmotic alterations (by adding 1.5% w/v of NaCl) in either inner W_1_ phase or outer W_2_ phase caused significant (*P* < 0.001) changes in W_1_ diameter whereby an increase in diameter was observed for samples prepared under hypo-osmotic (NaCl in W_1_ phase) conditions whereas a reduction in diameter was observed for samples under hyper-osmotic (NaCl in W_2_ phase) conditions. Change in droplet diameter was minimized at high concentration of Tween 80 (5%) compared to low concentration in Tween 80 (1%). Furthermore, a slight change in the diameter of the W_1_ droplet was observed during the first 30 minutes of the incubation period that increased rapidly after 60 minutes. No significant change in W_1_ droplet size was observed for samples without NaCl.

Oil globule measurements showed a significant (*P* < 0.001) reduction in droplet size at 30 minutes of incubation (approximately ∼22 μm reduction from time 0) for all samples. Oil globule size remained the same at 60 and 180 minutes of incubation for samples containing 5% of Tween 80 while an increase in droplet size was observed for samples containing 1% of Tween 80, with or without NaCl. The effect of different NaCl concentration on the increase (Fig. 5A) and reduction (Fig. 5B) of the inner W_1_ droplet diameter was determined as the release of bacteria depended on the stability of the inner W1 droplet. The increase (NaCl in inner W_1_ phase) and reduction (NaCl in outer W_2_ phase) in the diameter of the droplet was significant (*P* < 0.001) at high NaCl concentration (2.0% w/v) and lower Tween 80 concentration (1% w/v). Overall, samples prepared under hypo-osmotic conditions were more stable compared to samples prepared under hyper-osmotic conditions. The presence of sodium chloride in the inner W_1_ phase acted as osmotic regulators that counterbalanced the Laplace pressure whereas the presence of NaCl in the W_2_ phase resulted in a reduced osmotic pressure in the W_1_ phase unable to withstand the Laplace pressure.^37^

The data from Table S4† reveal that no significant (*P* = 1.00) change in size was observed for both inner W_1_ droplet and oil globule immediately after droplet formation with or without osmotic pressure alterations (during the first 30 minutes). A significant change (*P* < 0.001) in inner W_1_ droplet size was only observed immediately after the breakup/split of the W/O/W droplet releasing the inner W_1_ droplet into the W_2_ phase and forming a smaller oil globule or O/W droplet. The change in the W_1_ droplet occurred rapidly after droplet splitting as the escaped W_1_ droplet was only enclosed by a very thin film. As the splitting caused the formation of smaller oil globule or O/W droplet, a significant reduction (*P* < 0.001) in oil globule size was observed immediately after the splitting process (at 30 minutes) that remains stable afterwards. The absence of inner W_1_ phase in the oil globule after the splitting process caused the droplet to be unaffected by the change in osmotic pressure. A slight increase in oil globules size for samples containing low Tween 80 concentration mainly due to the undesirable interactions between the Tween 80 and the NaCl that weakens the interfacial strength and resulted in coalescence between neighbouring droplet.^37,40^

The stability of the W/O/W droplet depends on the osmotic and Laplace pressure balance.^41^ The main cause for droplet destabilization is attributed to the diffusion of water between the aqueous phases of the W/O/W emulsion due to osmotic imbalance as an extensive change in droplet size may cause bursting of the droplet releasing encapsulated contents into the outer continuous phase.^4^ The stability of the droplet against bursting depends on the interfacial strength and thickness along with the characteristics of the continuous outer phase such as the viscosity.^42^ Moreover, under hypo-osmotic conditions, the increase in NaCl concentration caused extensive swelling of the W_1_ droplet leading to droplet rupture as the film that enclosed the W_1_ droplet reaches its critical thickness.^42,43^ Reduction in the thickness of the interfacial film also lowers the activation energy needed to destabilize the droplet.^42^

The shrinkage of W_1_ droplet suspended in hyper-osmotic conditions is due to the migration of water from W_1_ towards W_2_ phase. The W_1_ droplet size reduction was accelerated after the splitting of W/O/W emulsion droplet as the splitting process resulted in the loss of the thick oil layer that functions as a barrier between the W_1_ and the W_2_ phase. By altering the osmotic balance between the inner and outer phase, it triggers the water flux through the thin film that is assisted by the surfactant molecules. When the W_1_ phase is closely in contact with the W_2_ phase of high solute concentration and separated by a very thin film, the water transport is mainly controlled by the hydrated-surfactant mechanism.^44^ During this process, the surfactant molecules hydrate at the O/W interface and migrate through the film to dehydrate at the W/O interface. Previously it was suggested that the movement of surfactant molecules through the oil phase to hydrate at the W/O interface and dehydrate at the O/W interface can weaken the interfacial film holding the emulsion droplet and lead to its collapse.^14^ However, more work is required to understand the mechanism that leads to emulsion droplet splitting.

Therefore, the stability of W_1_ droplet and oil globules in both hypo-osmotic and hyper-osmotic conditions is highly dependent on the NaCl and Tween 80 concentrations as they affect the integrity of the interfacial film. The release of bacteria is highly dependent on the stability of the inner W_1_ droplet whereby the presence of very thin film separating the inner W_1_ phase from the W_2_ phase after the splitting of W/O/W droplet making it more susceptible to droplet destabilization and bursting.

### 3.4 The effect of osmotic imbalances on the creaming behaviour

The thickness of the cream layer in the presence of different NaCl and Tween 80 concentrations was measured for both samples under hypo-osmotic and hyper-osmotic conditions. As the presence of bacteria in the W_1_ phase does not affect the change in droplet size, only samples containing bacteria were tested for creaming behaviour as presented in Fig. 7.

The change in creaming behaviour was dependent on NaCl and Tween 80 concentrations. A significant (*P* < 0.001) increase and reduction in creaming thickness were observed for sample prepared under hypo-osmotic condition (Fig. 7A) and hyper-osmotic condition (Fig. 7B) respectively with high concentration of NaCl and low concentration of Tween 80 at 180 minutes of storage time as compared to time 0. Change in creaming thickness was only observed at 60 minutes of incubation for sample containing low concentration of NaCl (0.5% w/v). As discussed previously, changes in the size of W_1_ droplets and oil globule occurred rapidly after the W/O/W emulsion droplet breakup/split. For oil globule, no significant (*P* = 1.00) change in oil globule size was observed after 60 to 180 minutes of the splitting process for samples containing 5% Tween 80 and lower NaCl concentration while an increase in size was observed for samples with 1% Tween 80 and higher NaCl concentrations. According to previous studies,^14,15^ under hypo-osmotic conditions, the increase in creaming thickness is attributed to the migration of water from the W_2_ to the W_1_ phase. However, for samples under hyper-osmotic conditions, the reduction in creaming thickness is attributed to the loss in oil globule from the W/O/W droplets.

For samples under hypo-osmotic conditions, at high NaCl concentration (2%), a significant increase (*P* < 0.001) in creaming thickness was observed at 30 to 60 minutes of incubation (Fig. 7A). However, a decrease in creaming size was observed after 180 minutes of the incubation period. Immediately after droplet splitting, the migration of water from W_1_ to W_2_ phase occurred rapidly and slowed down with a reduction in osmotic pressure gradient between the phases. Droplet bursting due to extensive swelling, in particular for samples containing a higher concentration of NaCl, resulted in the loss of the oil globule and a decrease in creaming. In addition, the reduction in interfacial strength due to high NaCl concentration^37,40^ caused coalescence between the oil globule forming a separated layer of oil on top of the samples, and a decrease in the creaming volume. Under hyper-osmotic condition (Fig. 7B), the loss in the cream layer is mainly attributed to the shrinkage of W_1_ droplets that diminished with a reduction in osmotic pressure gradient between the phases. Further loss in cream layer occurred due to bursting of W_1_ droplets and the coalescence between oil globules due to the undesirable interaction between NaCl and Tween 80 that weakens the interfacial strength of the droplet.

## 4. Conclusion

The current study demonstrates that the encapsulation of bacteria in W/O/W emulsion droplets improves their viability during storage. The presence of nutrients in the continuous aqueous phase promotes the growth of bacteria by the diffusion of nutrients to the dispersed aqueous phase through the oil layer. Moreover, the encapsulation of bacteria caused changes in their metabolic activity as the encapsulated cells showed higher glucose consumption compared to non-encapsulated cells. Therefore, W/O/W emulsion droplets have the potential to act as microreactors that can support the growth and metabolic activity of encapsulated bacterial cells. The optimisation of W/O/W emulsion release properties can be achieved through manipulating its stability and composition. Under osmotic balance alteration (hyper- and hypo-osmotic conditions), bacterial release from W/O/W emulsion can be modulated by changing salt or surfactant concentration. Furthermore, droplet breakup or splitting accelerates the release of bacteria with osmotic balance alterations. More work is still required to further understand the controlled release of bacteria from W/O/W droplet such as the mechanism behind the emulsion droplet breakup/split in order to improve the control of this process.

## Conflicts of interest

There are no conflicts of interest to declare.

## Supplementary Material

RA-011-D0RA10954A-s001
