# Supplementary material for: Optimisation of bacterial release from a stable microfluidic-generated water-in-oil-in-water emulsion
Source: RSC Adv. 2021 Feb 17;11(13):7738–49. doi: 10.1039/d0ra10954a (PMC8695039; doi:10.1039/d0ra10954a)
Supplement: RA-011-D0RA10954A-s001 [file RA-011-D0RA10954A-s001.pdf]

Electronic Supplementary Material (ESI) for RSC Advances.
This journal is © The Royal Society of Chemistry 2021

Optimisation of bacterial release from a stable microfluidic-generated water-in-oil-in-water emulsion

Nur Suaidah Mohd Isa[1,2], Hani El Kadri[2], Daniele Vigolo[2,3*], Konstantinos Gkatzionis[2,4*]

[1] Faculty of Fisheries and Food Science, Universiti Malaysia Terengganu, 21030 Kuala Terengganu, Terengganu, Malaysia

[2] School of Chemical Engineering, University of Birmingham, Birmingham, B15 2TT, UK

[3] School of Biomedical Engineering, University of Sydney, NSW 2006, Australia

[4] Department of Food Science and Nutrition, School of the Environment, University of the Aegean, Metropolite Ioakeim 2, 81400, Myrina, Lemnos, Greece

*Corresponding authors: kgkatzionis@aegean.gr; daniele.vigolo@sydney.edu.au

Supplementary information

A

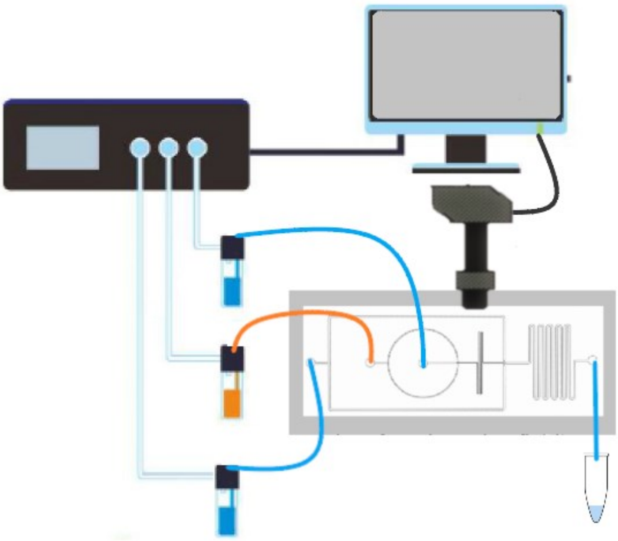

B

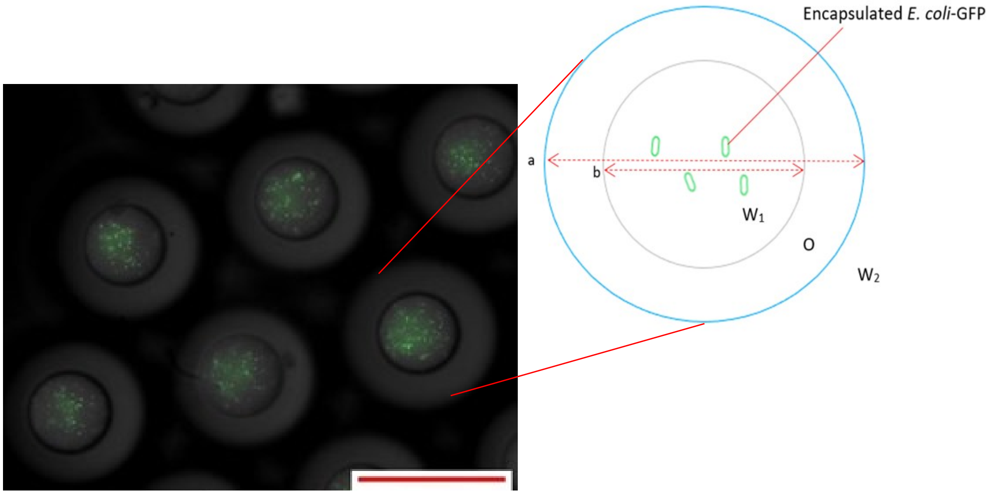

Figure S1. (A) Schematic representation of the microfluidic encapsulation whereby the different phases of W_1, oil phase and W_2 were flushed through the inlet by using a pressure controller. The produced $W_1/O/W_2$ droplet were collected continuously at the outlet. (B) Image and schematic representation of the $W_1/O/W_2$ droplet containing *E. coli*-GFP. Droplet formation was monitored by using a microscope with FASTCAM. The diameter of the oil globule is given by a: 100 µm while the diameter of the inner W_1 phase is given by b: 50 µm. Scale bar represents 100 µm.

Table S1. The encapsulation efficiency of *E. coli*-GFP in $W_1/O/W_2$ emulsions. Data represent mean ± standard deviation taken from 3 independent experiments.

**Formulation**	**Encapsulation efficiency (%)**
E. coli/DI water in W_1 (1% Tween 80)	99.99 ± 0.01
E. coli/DI water in W_1 (5% Tween 80)	100
E. coli/ LB broth in W_1 (1% Tween 80)	99.98 ± 0.02
E. coli/LB broth in W_1 (5% Tween 80)	99.98 ± 0.02
E. coli/ 0.5% NaCl in W_1 (1% Tween 80)	99.95 ± 0.02
E. coli/ 0.5% NaCl in W_1 (5% Tween 80)	99.97 ± 0.03

Table S2. The effect of different LB broth formulations on the viability of *E. coli*-GFP (Log CFU/mL). Data represent mean standard deviation from 3 independent experiments.

**Incubation time (hour)**	**Sodium chloride (0.5% w/v)**	**Tryptone**	**Yeast extract**
0	6.27 ± 0.05	6.40 ± 0.04	7.03 ± 0.09
24	6.18 ± 0.03	7.98 ± 0.03	8.26 ± 0.03
Change in Log CFU/mL	-1.48	24.72	17.48

Table S3. Average density measurements for the aqueous and oil phase of W/O/W emulsions at ambient temperature (25°C). The density for each sample was determined by measuring the mass per volume for each sample. Data represent mean ± standard deviation taken from 3 independent experiments.

**Solutions**	**Average density (g/mL)**
DIW	0.997 ± 0.001
DIW with *E. coli*-GFP	0.973 ± 0.001
DIW with 1% w/v Tween 80	0.986 ± 0.002
DIW with 5% w/v Tween 80	0.994 ± 0.001
Mineral Oil	0.863 ± 0.001
Mineral Oil with 1.5% w/v PGPR	0.849 ± 0.003
0.5% w/v NaCl	1.004 ± 0.002
1.5% w/v NaCl	1.012 ± 0.003
2.0% w/v NaCl	1.011 ± 0.002
0.5% w/v NaCl with *E. coli*-GFP	1.004 ± 0.002
0.5% w/v NaCl w 1% w/v Tween 80	1.011 ± 0.003
0.5% w/v NaCl with 5% w/v Tween 80	1.023 ± 0.004

Table S4. Change in mean W1 droplet and oil globule diameter (µm) at 30, 60 and 180 minutes with respect to 0 minutes of incubation. Samples were prepared in the presence or absence of E. coli-GFP in the W1 phase, with or without 1.5% w/v of NaCl in either W1 or W2 phase. Surfactant concentration was set at either 1% w/v or 5% w/v for Tween 80 in W2 phase. Data represent mean ± standard deviation from 3 independent experiments with N=900 droplet. The mean diameters were compared between samples within each incubation time (small letters) and between different incubation times within each sample (capital letters).

	**$W_1/O/W_2$ samples**	**30 minutes (µm)**		**60 minutes (µm)**		**180 minutes (µm)**	
		W_1	Oil Globule	W_1	Oil globule	W_1	Oil Globule
**With *E. coli*-GFP**	No NaCl, 1% Tween 80	0.015^{aA} ± 0.001	-22.25^{aA} ± 1.10	0.036^{aA} ± 0.002	-22.23^{aA} ± 1.301	0.051^{aA} ± 0.002	-19.99^{aA} ± 1.241
	No NaCl, 5% Tween 80	0.017^{aA} ± 0.001	-22.26^{aA} ±0.802	0.027^{aA} ± 0.001	-22.25^{aA} ± 0.913	0.032^{aA} ± 0.002	-22.23^{aA} ± 0.921
	1.5% NaCl in W_1, 1% Tween 80	0.947^{bA} ± 0.10	-22.24^{aA} ± 1.406	9.085^{bB} ± 0.62	-22.23^{aA} ± 0.923	15.304^{bC} ± 1.08	-18.04^{bB} ± 1.01
	1.5% NaCl in W_1, 5% Tween 80	0.317^{cA} ± 0.03	-22.256^{aA} ± 1.302	5.316^{cB} ± 0.51	-22.232^{aA} ± 1.311	9.865^{cC} ± 1.16	-22.22^{aA} ± 1.409
	1.5% NaCl in W_2, 1% Tween 80	-0.813^{dA} ± 0.06	-22.23^{aA} ± 1.105	-15.388^{dB} ± 1.02	-22.16^{aA} ± 0.805	-20.758^{dC} ± 1.04	-16.12^{cB} ± 0.903
	1.5% NaCl in W_2, 5% Tween 80	-0.381^{eA} ± 0.02	-22.23^{aA} ± 1.007	-6.732^{eB} ± 0.82	-22.23^{aA} ± 1.106	-10.534^{eC} ± 1.47	-22.2^{aA} ± 1.202
**Without *E. coli*-GFP**	No NaCl, 1% Tween 80	0.03^{aA} ± 0.002	-22.23^{aA} ± 0.801	0.038^{aA} ± 0.001	-22.23^{aA} ± 0.821	0.065^{aA} ± 0.002	-17.76^{aB} ± 0.701
	No NaCl, 5% Tween 80	0.025^{aA} ± 0.003	-22.24^{aA} ± 0.935	0.04^{aA} ± 0.004	-22.23^{aA} ± 0.905	0.048^{aA} ± 0.01	-22.22^{bA} ± 1.408
	1.5% NaCl in W_1, 1% Tween 80	1.021^{bA} ± 0.02	-22.22^{aA} ± 1.401	10.242^{bB} ± 1.62	-22.23^{aA} ± 1.207	16.527^{bC} ± 1.62	-16.98^{cB} ± 1.301
	1.5% NaCl in W_1, 5% Tween 80	0.439^{cA} ± 0.06	-22.23^{aA} ± 1.611	5.837^{cB} ± 1.13	-22.23^{aA} ± 1.503	10.104^{cC} ± 1.16	-22.22^{bA} ± 1.371
	1.5% NaCl in W_2, 1% Tween 80	-0.92^{dA} ± 0.12	-22.23^{aA} ± 0.781	-13.323^{dB} ± 0.91	-22.13^{aA} ± 0.838	-18.906^{dC} ± 1.02	-15.28^{dB} ± 0.911
	1.5% NaCl in W_2, 5% Tween 80	-0.368^{eA} ± 0.05	-22.22^{aA} ± 1.108	-9.048^{eB} ± 0.56	-22.22^{aA} ± 1.265	-13.015^{eC} ± 1.14	-22.2^{bA} ± 1.153

Data were analysed with one-way ANOVA.

^{abcde}means ± standard deviation and ^{ABC}means ± standard deviation with different letters are significantly different at $P < 0.05$.
